# Clinicopathologic study of E-cadherin/beta-catenin complex, and topoisomerase-II in a series of 71 liposarcoma cases

**DOI:** 10.1186/1477-7819-10-28

**Published:** 2012-02-02

**Authors:** Pinelopi Gogou, Emilios Pakos, Anna Batistatou, Ioannis Panelos, Evangelos Briasoulis, Dimitrios Stefanou, Nikoforos Apostolikas, Periclis Tsekeris

**Affiliations:** 1 Department of Radiation Oncology, University Ioannina, Medical School, Stavrou Niarhou Av 1., 45500 Ioannina, Greece; 2 Department of Pathology, University of Ioannina, Medical School, Ioannina, Greece, Stavrou Niarhou Av 1., 45500 Ioannina, Greece; 3 Department of Pathology, St Savvas Cancer Hospital, Athens, Greece; 4 Cancer Biobank Center, University of Ioannina, University Campus P.O. Box 1186, 45110 Ioannina, Greece

**Keywords:** liposarcomas, E-cadherin, b-catenin, topoisomerase II alpha, prognosis

## Abstract

**Background:**

To investigate the expression of E-cadherin, beta-catenin and topoisomerase-II alpha and examine their clinical relevance in liposarcomas.

**Materials and methods:**

The expression of E-cadherin, beta-catenin and topoisomerase II alpha was examined immunohistochemically on formalin-fixed paraffin-embedded tissue specimens from 71 patients who underwent surgical treatment for liposarcomas of the extremities or the retroperitoneum in two major cancer reference centres between 1990 and 2000. Detailed medical notes were available for all patients who were followed for median 82 months (range 5 to 215 months). Obtained expression data were weighted against clinical and pathology parameters of clinical relevance.

**Results:**

Patients were mostly male (59%), median age was 56 years for the liposarcomas of the extremities and 60 years for the retroperitoneal liposarcomas. The tumours were of diverse histology, grade and size (median diameters 7 and 17 cm for tumours of the extremities and retroperitoneum respectively). Expression of β-catenin protein was weakly detected in 15 cases (21.1%). Similarly weak expression of topoisomerase II-alpha was detected in 14 (19.7%) cases of which only two had more than 20% of tumor cells stained positive. E-cadherin was not detected in the studied cohort of liposarcomas. We did not detect associations between the expression of the above proteins by liposarcoma cells and clinical outcome.

**Conclusions:**

Liposarcomas do not express E-cadherin, which matches the absence of epithelioid differentiation in this sarcoma subtype, and have low topoisomerase II-alpha expression, which justifies to some extend their resistance to anthracycline-based chemotherapy.

## Background

Liposarcomas are the most common subtype of soft tissue sarcomas (STS) accounting for approximately 20% of all STS in adults [1,2]. The World Health Organization Committee classifies them in 5 subtypes according to the degree of differentiation [3]. Despite the fact that each histological subtype has a different clinical behavior and disease outcome, treatment is common for all liposarcoma subtypes and consists of wide resection of the tumor followed by additional radiotherapy and occasionally chemotherapy [4,5]. Although genetic tests have emerged in liposarcomas, still limited data exist regarding molecular profiling of these common STS subtypes [6].

The expression of E-cadherin/beta-catenin complex has been investigated in several tumors including STS [7]. The E-cadherin/beta-catenin complex is formed at cell-to-cell junctions and it is known to be involved in the wingless/Wnt signal transduction pathway. Wnt halts phosphorylation-degradation of the beta-catenin protein, which is consecutively accumulated in the cytoplasm and translocated to the nucleus where it functions as a transcription co-activator of several genes in involved in cell proliferation [8,9]. Interestingly, reduced expression of the E-cadherin/beta-catenin complex has been associated with aggressive tumor features such as poor differentiation, infiltrative growth, metastatic potential and short patient survival in several cancer types [10,11]. The DNA topoisomerase-II-alpha (TOP2α) is one of the major nuclear proteins with peak expression at G2/M phase. It is virtually involved in every aspect of DNA metabolism, playing an important role in chromosome organization and segregation [12]. This cellular molecule is considered a key modulator of anticancer activity of anthracycline drugs [13,14].

In the present study we evaluated the expression of E-cadherin, beta-catenin and TOP2α proteins in a series of 71 liposarcoma cases and investigated potential associations of these molecules with clinical outcome.

## Methods

Formalin-fixed paraffin-embedded tissue specimens from patients who had undergone surgical treatment for liposarcomas for whom detailed medical notes and adequate follow up records were made available, were selected for this study.

Two co-author pathologists reviewed tumor specimens, blinded to clinical information, at the Pathology Department of the Ioannina University Hospital. (B.A., & P.I.) Histological typing was based on WHO classification of soft tissue tumors.

Immunostaining was performed on formalin-fixed, paraffin-embedded tissue sections using the EnVision System (DAKO Corp, Netherlands), and the monoclonal antibodies: E-cadherin (CM170B, Biocare Medical, California), beta-catenin (DBS, Menarini, Hellas) and DNA topoisomarase II-alpha (Ki-S1, DAKO). The immunohistochemical (IHC) evaluations were performed as previously described [15]. The evaluation of IHC detected expression of E-cadherin, beta-catenin and TOP2α was performed by a semiquantitative method. The expression of each studied protein was considered "weak" if 1% to 20% of cancer cells were stained immunohistochemically, "moderate" if 21% to 50% were stained and "strong" if more than 50% of cancer cells stained. Nuclear and cytoplasmic staining for beta-catenin and TOP2α were evaluated separately. Also, we included the intensity of staining in the classification of the each protein. Expression of each one of the proteins was investigated for association with clinical and pathological parameters, such as grade, subtype, location, grade, surgical margins, relapse, metastatic potential and overall survival. For the purposes of the correlative analysis we used 20% stained tumor cells as a cut-off level, above which, protein expression was considered positive.

### Statistical Analysis

The expression of E-cadherin, beta-catenin, TOP2α was investigated for associations with various pathological and clinical variables. Fisher's test and Cox models estimated hazard ratios were used to evaluate each candidate predictor. P values of < 0.05 were considered statistically significant; all p values were two-tailed. All the dates were calculated from the day of diagnosis. Statistical analyses were performed by using the Statistical Program SPSS 14. (Chicago, IL, USA).

## Results

### Demographics

**Figure 1:**
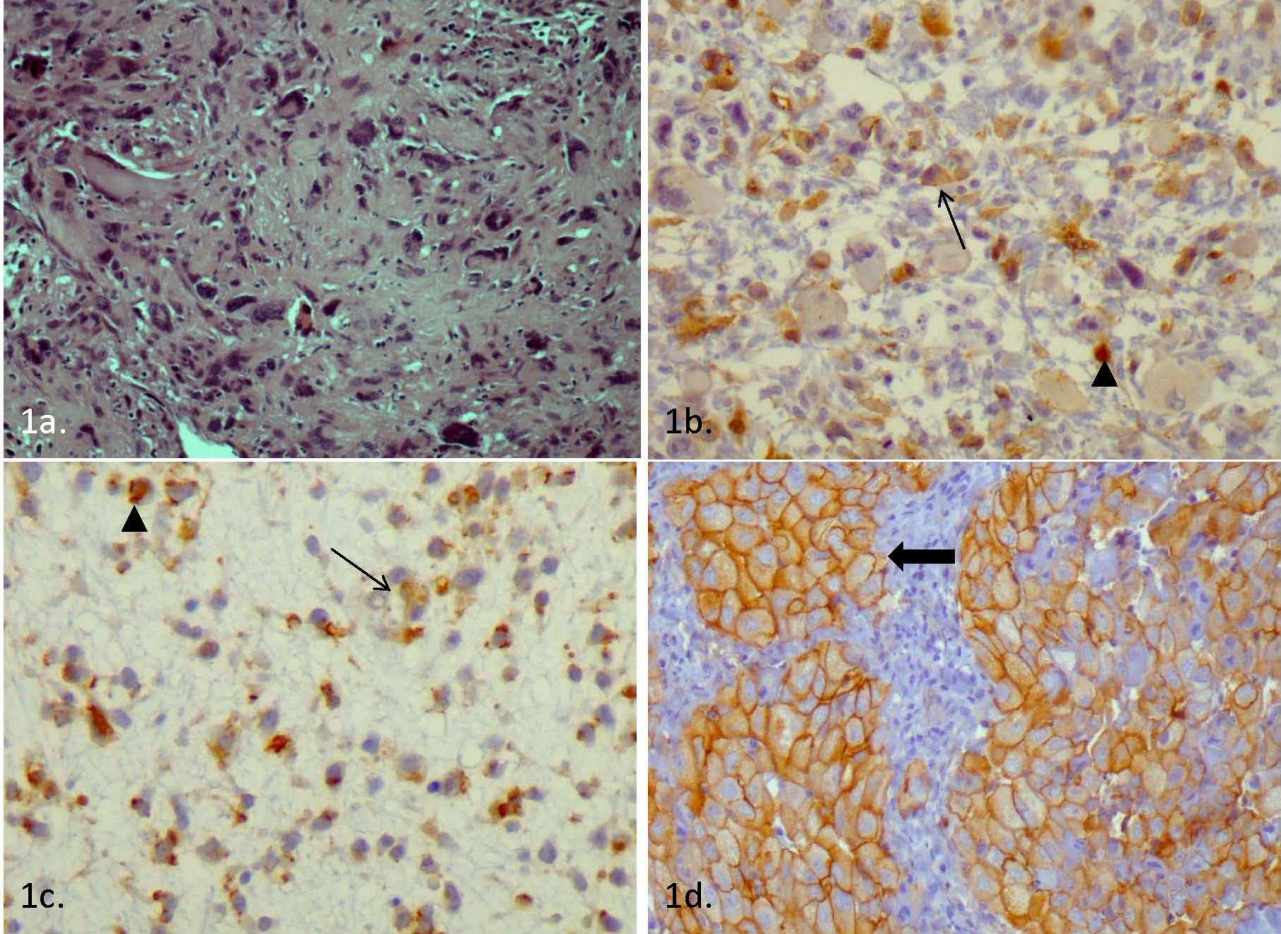
**a) Retroperitoneal pleomorphic liposarcoma (H&E x400) b) Weak expression of beta-catenin in pleomorphic retroperitoneum liposarcoma (< 20% of neoplastic cells showed cytoplasmic immunoreactivity (*long arrow*) and < 5% nuclear immunoreactivity (*short arrow*) (DAB X400))**. c) Weak expression of beta-catenin in extremity liposarcoma ((< 20% of neoplastic cells showed cytoplasmic immunoreactivity (*long arrow*) and < 5% nuclear immunoreactivity (*short arrow*) (DAB x400). d) Area with extensive membranous and cytoplasmic expression of beta-catenin in an extremity liposarcoma ((< 50% of neoplastic cells showed cytoplasmic and membranous immunoreactivity (*long arrow*) (DAB x400)).

A total of 71 liposarcoma patients were included in the study. The median age of patients was 56 years (range 20-86), and 42 were males (59%). Fifty-five liposarcomas were located in the extremities, and 16 were retroperitoneal. They had diverse histological liposarcoma subtypes. In the extremities and the retropetinoneum the commonest histological subtype was the well differentiated. In the extremities the commonest localization was the thigh. (Figure 1c,d)

All patients received operation by different surgeons on curative intent; surgical excision was performed in the majority (68 patients) and 3 patients underwent therapeutic limb amputation. Twenty-four patients had positive surgical margins. All patients with positive surgical margins (24) and others with marginal resection and/or high grade characteristics were given adjuvant postoperative therapy. In all, 47 patients received adjuvant radiotherapy and 11 patients adjuvant chemotherapy. Patient characteristics are presented in Table 1.

**Table 1:** Demographics (percentages were calculated separately in each category)

	EXTREMITIES (N = 55)	RETROPERITONEUM (N = 16)
**Age: median (range)**	56 (31-80 years)	64 (37-86 years)
**N Males (%)**	32(58%)	10(63%)
**Median Size in mm (range)**	70.0 (7-280)	170.0 (35-500)
**Location (%)**		
*Thigh*	27 (49%)	-
*Trunk*	14 (26%)	-
*Leg*	6 (11%)	-
*Arm*	4 (7%)	-
*Forearm*	3 (5%)	-
*Hand*	1 (2%)	-
*Retroperitoneum*	-	16 (100%)
**Histological subtype (%)**		
*Well-differantiated liposarcomas with myxoid stroma*	32 (58%)	10 (62%)
*Pleomorhic*	19 (35%)	1 (7%)
*Round cell*	2 (3%)	2 (12%)
*Sclerosing well-differentiated*	1(2%)	2 (12%)
*Well-differentiated Lipoma-like/adipocyticliposarcoma*	1 (2%)	1 (7%)
**Grade**		
I *(low)*	38 (69%)	12 (75%)
II-III *(High)*	17 (31%)	4 (25%)
**Type of surgery N (%)**		
*Surgical excision*	52(94%)	16(100%)
*Amputation*	3(6%)	0(0%)
**Surgical Margins**		
*Positive*	16 (29%)	8(50%)
*Negative*	39 (71%)	8(50%)
**Chemotherapy**		
*Yes*	7(12%)	4(25%)
*No*	48(88%)	12(75%)
**Radiotherapy**		
*Yes*	36(66%)	11(69%)
*No*	19(34%)	5(31%)
**Median total radiation dose (range)**	57.0 Gy (20.0-64.0)	40.0 Gy (40.0-64.0)
		

The median follow up of patients was 82 months (range 5 to 215 months). Within this follow-up period 54 patients died. Thirty eight patients (53,5%) developed local recurrences and another 16, metastatic disease (22,5%). Patients with retroperitoneal liposarcomas had higher local recurrence and death rates compared to those with tumor localization in the extremities (Table 1).

### Expression of E-cadherin, beta-catenin and topoisomerase II alpha (Table 2)

Immunostaining for E-cadherin was negative in all cohort cases. Beta-catenin expression was documented in 15 liposarcomas (27.3%). In 13 cases beta-catenin was weakly expressed (1% to 10% stained tumor cells) (Figure 1b,c) and in 2 it was moderate (40% and 50% of tumor cells stained) (Figure 1d). In the majority of cases beta-catenin was found located at the membrane (12/15). The intensity of beta-catenin was characterized weak and moderate in 7 and 8 sarcomas, respectively.

**Figure 2:**
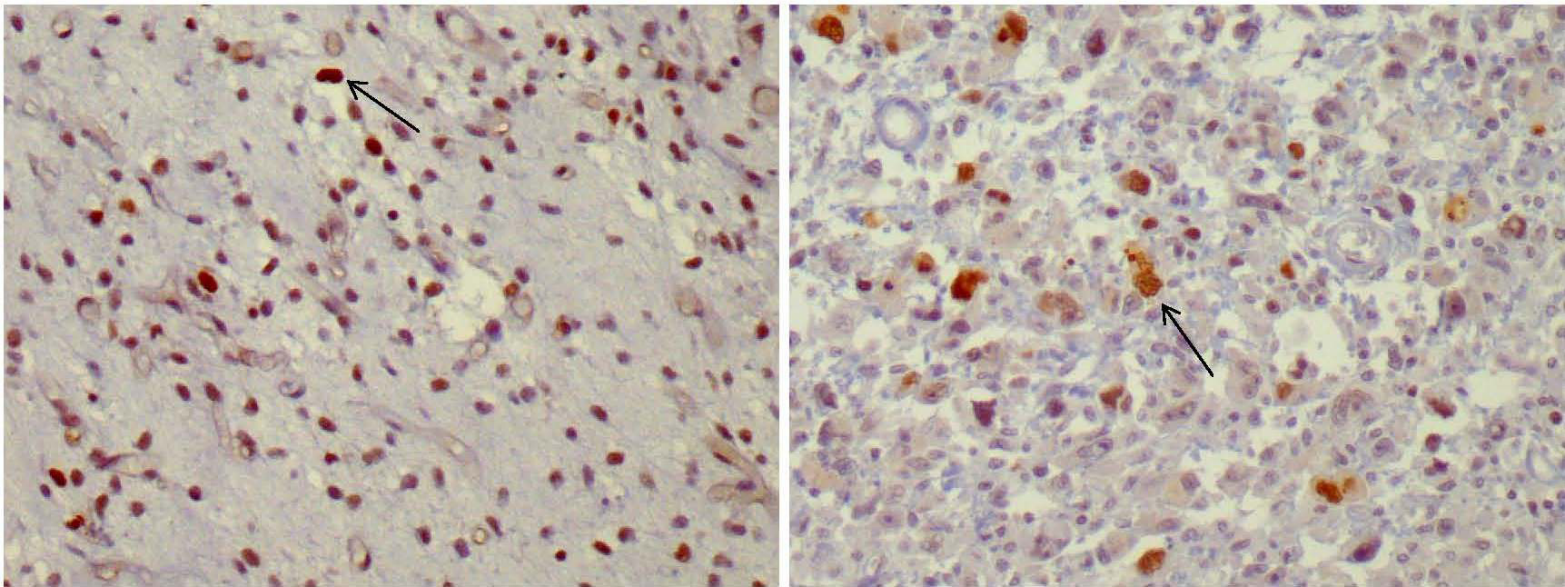
**a) Area of extremity liposarcoma with moderate expression of topoisomerase IIa (21-50% of neoplastic cells showed nuclear immunoreactivity (*arrow*) (DAB X400)**. b) Weak expression of topoisomerase IIa in extremity liposarcoma (< 20% of neoplastic cells showed nuclear immunoreactivity (*arrow*) (DAB X400).

TOP2α expression was detected in 14 cases. This was moderate in 2 extremity liposarcomas (Figure 2a) and weak in all other cases (Figure 2b). No strong TOP2α intensity was observed. In all tumors that expressed TOP2α, the molecule had nuclear location. The expression of the molecules in each subgroup (extremities, retroperitoneum) is presented in Table 2. We did not observe any statistically significant differences in the expression of beta-catenin and TOP2α between the extremities and the retroperitoneum (Table 2).

**Table 2:** Expression of E-cadherin, β-catenin and topoisomerase IIalpha proteins detected by IHC in 71 liposarcomas.

	EXTREMITIES	RETROPERITONEUM	*p *Value
E-Cadherin expression			
*No expression*	55 (100%)	16 (100%)	NS
Beta-catenin expression			
No expression	43 (78.2%)	13 (81.2%)	NS
*1%-20%*	10 (18.2%)	3 (18.8%)	NS
*21-50%*	2 (3.6%)	0 (0%)	NS
*>50%*	0 (0%)	0 (0%)	NS
Beta-catenin location			
*Membranous*	11	1	0.08
*Nuclear*	1	2	NS
Beta Catenin intensity			
*Moderate*	7	1	NS
*Weak*	5	2	NS
Topoisomerase IIa expression			
*No expression*	45 (81.9%)	12 (75%)	NS
*1%-20%*	8 (14.5%)	4 (25.0%)	NS
*21-50%*	2 (3.6%)	0 (0%)	NS
*>50%*	0 (0%)	0 (0%)	NS
Topoisomerase IIa location			
*Membranous*	0	0	NS
*Nuclear*	10	4	NS
Topoisomerase IIa intensity			
*Moderate*	8	2	NS
*Weak*	2	2	NS

P value is referring in the comparison between the extremities and retroperitoneum by using Fisher's test

NS: non-significant (p>0.05).

### Clinicopathologic associations of beta-catenin and topoisomerase II alpha

No correlation was found between expression of beta-catenin or TOP2α and pathological factors, such as tumor grade and histological subtype.

Beta-catenin (either membranous or nuclear expression) was not associated with local recurrences (p = 0.67), metastases (p = 0.47) or death (p = 0.47). Similarly, TOP2α was not associated with clinical outcome (p values of 0.52, 0.57 and 0.78 for local recurrences, metastases and death, respectively). The cut-off level of expression in more than 20% of tumor cells was used in the pertinent analyses. However, different cut-off levels were also utilized (i.e. any expression) with no change in the results. Finally, similar results were obtained when the analyses were performed separately in extremities and retroperitoneal liposarcomas.

## Discussion

Liposarcomas, is one of the commonest soft tissue sarcomas, but have been poorly investigated regarding their molecular profile. We evaluated the expression of E-cadherin, beta-catenin and TOP2α in a cohort of 71 liposarcoma cases and investigated for possible associations with pathological characteristics and clinical outcome. This is, to our knowledge, the largest study that investigated E-cadherin, beta-catenin and topsiomerase II alpha in liposarcomas.

We did not detect expression of E-cadherin in our series, while 21% of cases were found to express beta-catenin and 20% TOP2α. We consider that absence of E-cadherin expression signifies the apparent mesenchymal origin of liposarcomas and indicates lack of any degree of epithelial differentiation in these tumors [7]. Similarly, other investigators have also reported lack of expression of E-cadherin in smaller series of liposarcomas [7,16].

Regarding beta-catenin, few studies have investigated its expression in liposarcomas. Ng et al., found only 2 of 31 liposarcomas with increased beta-catenin [17] and Sakamoto et al., reported only cytoplasmic expression in 5 out of 12 studied cases [18]. However, none of these studies reported membranous beta-catenin expression, which prevailed in our series. In our study 3 cases (4.2%) had weak expression of beta-catenin localized in the nucleus and 15% in the membrane. Although membranous beta-catenin expression was detected in only a small percentage of studied cohort this is still higher than the percentage reported in previous studies. This finding indicate that some liposarcomas may utilize at least in part beta-catenin cell to cell adhesion. Nuclear accumulation of beta-catenin is known to be involved in the Wnt signalling pathway and interplay of these two proteins have been implicated in several human carcers and in aggressive synovial sarcomas [19,20]. However this usually involves strong nuclear expression, which was not the case in our series [20]. A major finding in our study was the predominately membranous localization of beta-catenin, which among STS has only been described in uterine leiomyosarcomas [21]. Since, membranous beta-catenin expression was low, if any, in our liposarcomas, definite conclusion about its association with the low metastatic potential of the majority of these tumors can not be drawn [22,23].

The investigation of TOP2α in sarcomas has already drawn the attention of several investigators and our group [15,24]. This is due to its significant biologic role in regulating DNA metabolism and function and also because this enzyme is the target of the anthracyclin doxorubicin, which is the main chemotherapy drug with activity in sarcomas. The presence of TOP2α is considered a prerequisite for anthracyclins to exert their cytotoxic effects given that the activity of these drugs correlates the nuclear content of the enzyme [14,25]. In addition high expression of TOP2α has been profilied as an indicator of tumor aggressiveness and poor outcome in several tumor types [26].

It must be noted herein that, Endo et al. found DNA TOP2α to be intensively expressed in cell contours in mature adipocytes and lipoblasts in all benign and malignant lipomatous tumors, which led them suggest that membranous immunostaining for TOP2α might be a useful marker for diagnosing liposarcoma [27]. However we did not detect membranous localisation of TOP2α but only weak nuclear expression. We consider that faint nuclear TOP2α expression in our series associates well to the limited activity of anthracyclins in liposarcomas and also the relatively favourable prognosis of this sarcoma subtype which still remains dismal [5,28].

## Conclusion

Profiling of the expression of three studied molecules in this study elucidates to some extent some key clinical aspects of liposarcomas: lack of E-cadherin expression verifies the mesenchymal origin and weak beta-catenin and TOP2α expression provide molecular reasoning of the limited aggressiveness and marginal chemosensitivity of these tumours.

## Competing interests

The authors declare that they have no competing interests.

## Authors' contributions

**PG: **carried out material and data acquisition, did literature search, drafted the manuscript. **EP: **participated in the design of the study and performed the statistical analysis **AB: **carried out the immunohistochemistry studies, and review mmanuscript. **IP: **carried out the immunohistochemistry studies **EB: **participated in study design, data interpretation drafted and edited the manuscript. **DS: **carried out the immunohistochemistry studies and drafted the manuscript. **NA: **carried out the immunohistochemistry studies **PT: **proposed, designed and coordinated the study. All authors read and approved the final manuscript.

## References

[B1] Coindre JM Pedeutour F Aurias A Well-differentiated and dedifferentiated liposarcomas Virchows Arch 2010 456 2 167 179 10.1007/s00428-009-0815-x 19688222

[B2] Clark MA Fisher C Judson I Thomas JM Soft-tissue sarcomas in adults N Engl J Med 2005 353 7 701 711 10.1056/NEJMra041866 16107623

[B3] Fletcher CD The evolving classification of soft tissue tumours: an update based on the new WHO classification Histopathology 2006 48 1 3 12 10.1111/j.1365-2559.2005.02284.x 16359532

[B4] Reitan JB Kaalhus O Radiotherapy of liposarcomas Br J Radiol 1980 53 634 969 975 10.1259/0007-1285-53-634-969 7426920

[B5] Karavasilis V Seddon BM Ashley S Al-Muderis O Fisher C Judson I Significant clinical benefit of first-line palliative chemotherapy in advanced soft-tissue sarcoma: retrospective analysis and identification of prognostic factors in 488 patients Cancer 2008 112 7 1585 1591 10.1002/cncr.23332 18278813

[B6] Fritz B Schubert F Wrobel G Schwaenen C Wessendorf S Nessling M Korz C Rieker RJ Montgomery K Kucherlapati R Microarray-based copy number and expression profiling in dedifferentiated and pleomorphic liposarcoma Cancer Res 2002 62 11 2993 2998 12036902

[B7] Sato H Hasegawa T Abe Y Sakai H Hirohashi S Expression of E-cadherin in bone and soft tissue sarcomas: a possible role in epithelial differentiation Hum Pathol 1999 30 11 1344 1349 10.1016/S0046-8177(99)90066-7 10571515

[B8] Moon RT Wnt/beta-catenin pathway Sci STKE 2005 2005 271 cm1 10.1126/stke.2712005cm1 15713948

[B9] Nelson WJ Nusse R Convergence of Wnt, beta-catenin, and cadherin pathways Science 2004 303 5663 1483 1487 10.1126/science.1094291 15001769 PMC3372896

[B10] Jeanes A Gottardi CJ Yap AS Cadherins and cancer: how does cadherin dysfunction promote tumor progression? Oncogene 2008 27 55 6920 6929 10.1038/onc.2008.343 19029934 PMC2745643

[B11] Kase S Sugio K Yamazaki K Okamoto T Yano T Sugimachi K Expression of E-cadherin and beta-catenin in human non-small cell lung cancer and the clinical significance Clin Cancer Res 2000 6 12 4789 4796 11156236

[B12] Nitiss JL DNA topoisomerase II and its growing repertoire of biological functions Nat Rev Cancer 2009 9 5 327 337 10.1038/nrc2608 19377505 PMC2730144

[B13] Esteva FJ Hortobagyi GN Topoisomerase II{alpha} amplification and anthracycline-based chemotherapy: the jury is still out J Clin Oncol 2009 27 21 3416 3417 10.1200/JCO.2009.22.6449 19470912

[B14] Nitiss JL Targeting DNA topoisomerase II in cancer chemotherapy Nat Rev Cancer 2009 9 5 338 350 10.1038/nrc2607 19377506 PMC2748742

[B15] Gogou PN Batistatou A Pakos EE Apostolikas N Stefanou D Tsekeris PG E-cadherin, b-catenin and topoisomerase II expression in rhabdomyosarcomas J BUON 2009 14 2 323 324 19650189

[B16] Yoo J Park S Kang CS Kang SJ Kim BK Expression of E-cadherin and p53 proteins in human soft tissue sarcomas Arch Pathol Lab Med 2002 126 1 33 38 11800644 10.5858/2002-126-0033-EOECAP

[B17] Ng TL Gown AM Barry TS Cheang MC Chan AK Turbin DA Hsu FD West RB Nielsen TO Nuclear beta-catenin in mesenchymal tumors Mod Pathol 2005 18 1 68 74 10.1038/modpathol.3800272 15375433

[B18] Sakamoto A Oda Y Adachi T Saito T Tamiya S Iwamoto Y Tsuneyoshi M Beta-catenin accumulation and gene mutation in exon 3 in dedifferentiated liposarcoma and malignant fibrous histiocytoma Arch Pathol Lab Med 2002 126 9 1071 1078 12204056 10.5858/2002-126-1071-CAAGMI

[B19] Clevers H Wnt/beta-catenin signaling in development and disease Cell 2006 127 3 469 480 10.1016/j.cell.2006.10.018 17081971

[B20] Hasegawa T Yokoyama R Matsuno Y Shimoda T Hirohashi S Prognostic significance of histologic grade and nuclear expression of beta-catenin in synovial sarcoma Hum Pathol 2001 32 3 257 263 10.1053/hupa.2001.22764 11274633

[B21] Abeler VM Royne O Thoresen S Danielsen HE Nesland JM Kristensen GB Uterine sarcomas in Norway. A histopathological and prognostic survey of a total population from 1970 to 2000 including 419 patients Histopathology 2009 54 3 355 364 10.1111/j.1365-2559.2009.03231.x 19236512

[B22] Hsu PK Li AF Wang YC Hsieh CC Huang MH Hsu WH Hsu HS Reduced membranous beta-catenin protein expression is associated with metastasis and poor prognosis in squamous cell carcinoma of the esophagus J Thorac Cardiovasc Surg 2008 135 5 1029 1035 10.1016/j.jtcvs.2007.11.007 18455580

[B23] Rosen DG Zhang Z Chang B Wang X Lin E Liu J Low membranous expression of beta-catenin and high mitotic count predict poor prognosis in endometrioid carcinoma of the ovary Mod Pathol 2010 23 1 113 122 10.1038/modpathol.2009.141 19820688

[B24] Gogou PN Batistatou A Pakos EE Apostolikas N Stefanou D Tsekeris PG Expression of E-cadherin, beta-catenin and topoisomerase IIalpha in leiomyosarcomas Clin Transl Oncol 2009 11 8 548 551 10.1007/s12094-009-0401-3 19661031

[B25] Chikamori K Grozav AG Kozuki T Grabowski D Ganapathi R Ganapathi MK DNA Topoisomerase II Enzymes as Molecular Targets for Cancer Chemotherapy Curr Cancer Drug Targets 2010 10.2174/156800910793605785 20578986

[B26] Rody A Karn T Ruckhaberle E Muller V Gehrmann M Solbach C Ahr A Gatje R Holtrich U Kaufmann M Gene expression of topoisomerase II alpha (TOP2A) by microarray analysis is highly prognostic in estrogen receptor (ER) positive breast cancer Breast Cancer Res Treat 2009 113 3 457 466 10.1007/s10549-008-9964-x 18340528

[B27] Endo H Hirokawa M Ishimaru N Tanaka Y Yamashita M Sakaki M Hayashi Y Sano T Unique cell membrane expression of topoisomerase-II alpha as a useful diagnostic marker of liposarcoma Pathol Int 2004 54 3 145 150 10.1111/j.1440-1827.2003.01600.x 14989736

[B28] Krikelis D Judson I Role of chemotherapy in the management of soft tissue sarcomas Expert Rev Anticancer Ther 2010 10 2 249 260 10.1586/era.09.176 20132000

